# Distance from Home to Study Clinic and Risk of Follow-Up Interruption in a Cohort of HIV-1-Discordant Couples in Nairobi, Kenya

**DOI:** 10.1371/journal.pone.0043138

**Published:** 2012-08-21

**Authors:** N. Jeanne Conley, Patricia B. Pavlinac, Brandon L. Guthrie, Romel D. Mackelprang, Anthony N. Muiru, Robert Y. Choi, Rose Bosire, Ann Gatuguta, Carey Farquhar

**Affiliations:** 1 Department of Epidemiology, University of Washington, Seattle, Washington, United States of America; 2 Department of Medicine, University of Washington, Seattle, Washington, United States of America; 3 Department of Global Health, University of Washington, Seattle, Washington, United States of America; 4 Harvard Medical School, Boston, Massachusetts, United States of America; 5 Kenya Medical Research Institute, Nairobi, Kenya; 6 Department of Public Health, Kenyatta University, Nairobi, Kenya; Tulane University School of Public Health and Tropical Medicine, United States of America

## Abstract

**Background:**

Longitudinal studies of HIV-1-infected individuals or those at risk of infection are subject to missed study visits that may have negative consequences on the care of participants and can jeopardize study validity due to bias and loss of statistical power. Distance between participant residence and study clinic, as well as other socioeconomic and demographic factors, may contribute to interruptions in patient follow-up.

**Methods:**

HIV-1-serodiscordant couples were enrolled between May 2007 and October 2009 and followed for two years in Nairobi, Kenya. At baseline, demographic and home location information was collected and linear distance from each participant’s home to the study clinic was determined. Participants were asked to return to the study clinic for quarterly visits, with follow-up interruptions (FUI) defined as missing two consecutive visits. Cox proportional hazards regression was used to assess crude and adjusted associations between FUI and home-to-clinic distance, and other baseline characteristics.

**Results:**

Of 469 enrolled couples, 64% had a female HIV-1-infected partner. Overall incidence of FUI was 13.4 per 100 person-years (PY), with lower incidence of FUI in HIV-1-infected (10.8 per 100 PY) versus -uninfected individuals (16.1 per 100 PY) (hazard ratio [HR] = 0.66; 95% confidence interval [CI]: 0.50, 0.88). Among HIV-1-infected participants, those living between 5 and 10 kilometers (km) from the study clinic had a two-fold increased rate of FUI compared to those living <5 km away (HR = 2.17; 95% CI: 1.09, 4.34). Other factors associated with FUI included paying higher rent (HR = 1.67; 95% CI: 1.05, 2.65), having at least primary school education (HR = 1.96; 95% CI: 1.02, 3.70), and increased HIV-1 viral load (HR = 1.23 per log_10_ increase; 95% CI: 1.01, 1.51).

**Conclusions:**

Home-to-clinic distance, indicators of socioeconomic status, and markers of disease progression may affect compliance with study follow-up schedules. Retention strategies should focus on participants at greatest risk of FUI to ensure study validity.

## Introduction

Missing data resulting from participant attrition, or incomplete study follow-up, threaten the integrity of a study, with consequences ranging from a reduction in statistical power to significantly biased results and diminished validity. While numerous techniques have been developed to address the problem, none are simple, most require manipulation of the data, and the precision of results can be difficult to determine [1]–[7]. Missing data could be reduced or preemptively addressed by predicting which participants are most likely to miss visits or be lost to follow-up. Eligibility criteria can be designed to limit enrollment to those with more adherent profiles and supplemental support procedures can be implemented to improve contact with those most at risk of follow-up interruptions.

Predicting attrition is challenging and has been the subject of a number of studies in the U.S., Western Europe and sub-Saharan Africa. Reviews of published studies have listed up to 18 different definitions for patients who were considered lost to follow-up [8], [9], ranging from missed visits to complete disappearance from a study. Despite varying operational definitions of study attrition, certain study designs appear to be associated with higher attrition rates, specifically those that have a longer duration of follow-up [10] or frequent, complex or time-consuming visits [1], [11], [12]. The use of unskilled or inconsistently trained staff and the provision of limited incentives also lead to higher attrition [11], [13]. Participant characteristics also appear to play an important role in the likelihood of follow-up interruption. In the US, characteristics of study participants at risk for attrition include lower income [13], less education [13], black race [1], [13], lower CD4 count [1], [10], younger age [1], [10], [14], [15], hospitalization or incarceration [16], injection drug use [1], [10], [16], unwillingness to travel [16], and increased life stressors, multiple health problems and inconsistent use of health care resources [1], [11]. Gender differences in rates of missed visits have been inconclusive [1], [11], [13], [15].

In sub-Saharan Africa and other resource-limited settings, reasons for not completing a study are not fully understood. Despite the paucity of data on the subject, some factors have been associated with attrition in antiretroviral treatment (ART) programs in sub-Saharan Africa, including younger age [17]–[19], baseline CD4 cell count [18], [20]–[22] and male gender [17], [18], [20], [22], [23]. Many of the retention difficulties described by patients are similar to those listed by study participants in the United States, including health problems [17], [24], concurrent family and/or work commitments [25], [26], limited clinic hours [25], long appointments [25]–[27], lack of child care [8], and lost wages or employment [25], [26], [28]. Cultural or socio-economic factors may pose considerable difficulty for study participants with HIV in Africa. These include fear of disclosure of HIV status [27], [29], bias of family and friends against study participation [30], spousal abuse [29], stigma [26], [27], and the financial costs associated with the program [26]–[28], [31], [32], particularly those relating to transportation [24], [27], [29], [32], [33].

Distance-decay is used to explain how health care use is negatively correlated with the distance patients must travel to access care [34]–[36]. Specifically, distance-decay implies that patients who must travel greater distances may delay seeking health care and have worse outcomes [37]. Research studies often require multiple study visits at a higher frequency than routine care, and similar effects of travel distance on adherence to visit schedules may also occur in research settings. To examine this possibility and assess other correlates of poor adherence to study visits, we used follow-up records from a prospective cohort study of HIV-1-discordant couples conducted in Nairobi, Kenya to determine whether the distance between a participant’s home and the research clinic was an important factor in explaining missed study visits.

## Methods

### Ethics Statement

Written informed consent was obtained from all participants. This study received ethical approval from the institutional review board of the University of Washington and the Ethics and Research Committee of the University of Nairobi and was conducted according to the guidelines set forth by the United States Department of Health and Human Services.

### Recruitment and Enrollment

HIV-1-serodiscordant heterosexual couples were enrolled in a prospective cohort study between May 2007 and October 2009. Couples were eligible if one member of the couple was HIV-1-infected and the other HIV-1 susceptible, both partners reported having sexual intercourse at least 3 times in the previous 3 months, and planned to stay together for at least two years. Participants who were pregnant or using antiretroviral therapy at enrollment were excluded.

Couples were referred to the study from HIV voluntary counseling and testing (VCT) sites throughout the greater Nairobi region and from a clinical trial site at Kenyatta National Hospital that had identified discordant couples. Couples provided written informed consent at the screening and enrollment visits as well as contact and locator information for retention-tracking purposes. All couples received at least one home visit from a peer counselor to verify the location of their residence. Study clinicians collected baseline sociodemographic, sexual/reproductive and medical history data from both members of the couple, and blood and genital samples were collected at screening, enrollment and subsequent visits.

### Study Follow-up Visits

Couples were asked to return to the study clinic quarterly to provide blood and genital specimens, complete questionnaires on recent sexual behavior and current health status, and be tested for HIV-1 seroconversion. HIV-1-infected partners were supplied with trimethoprim-sulfamethoxazole to be taken daily as prophylaxis for opportunistic infections. Couples continued to be followed in the study even if they separated, the female partner became pregnant, or one partner left the study.

Support groups for those with and without HIV infection were available throughout the study. Drinks were supplied free of charge during clinic visits, and participants were reimbursed 500 Kenyan Shillings (KSh), (approximately 3.50 US dollars [USD]) per visit for transportation expenses to and from the clinic.

### Laboratory Procedures

At enrollment and quarterly visits, HIV status of the uninfected partner was determined by two rapid tests conducted in parallel using the Determine HIV-1/2 rapid test (Abbott Laboratories, Tokyo, Japan, now marketed by Inverness Medical as Alere Determine) and the Bioline HIV 1/2 rapid test (Standard Diagnostics Inc., Suwon, Korea), with confirmation by the Vironostika HIV Uni-form II Ag/Ab ELISA kit (bioMérieux SA, Marcy l’Etoile, France) for positives or indeterminates. Urine pregnancy tests were also performed every three months (Quick Vue One Step hCG Urine Pregnancy kit, Quidel Corp, San Diego, CA). CD4 cell counts (FACSCaliber, BS Bioscience, Franklin Lakes, USA) and HIV-1 RNA viral load (Gen-Probe Transcription Mediated Amplification, San Diego, USA) were measured in HIV-1-infected participants at enrollment and every six months.

### Geographic Mapping

The locations of participants’ residences were mapped using locator information provided by each participant. Based on this information, a member of the study retention team mapped each residence using a web-based mapping application built on the Google Maps engine (Google, Inc, Mountain View, CA). The geographic coordinates for all VCT sites and the study clinic were determined using a handheld global positioning system (GPS) unit (Trimbel Juno, Sunnyvale, USA). The resulting coordinates were used to estimate linear (as the crow flies) distances between each residence and the referring VCT and between each residence and the study clinic using a geospatial algorithm (Google, Inc).

**Table 1:** Baseline characteristics of study participants.

	Couples with HIV-1-Infected Male Partner		Couples with HIV-1-Infected Female Partner	
	HIV-1-Infected	HIV-1-Uninfected	HIV-1-Uninfected	HIV-1-infected
	Male	Female	Male	Female
	N = 168	N = 168	N = 301	N = 301
**Characteristic**	**median (IQR) or n (%)**			
Age (Years)	36 (32, 42.5)	29 (25, 35)	34 (29, 39)	28 (24, 34)
Relationship duration (years)	6.3 (2.4, 10.9)	5.8 (2.4, 11.2)	4.77 (2.4, 9.5)	4.8 (2.3, 8.7)
CD4 Count (cells/µl)	340.5 (231, 503)	–	–	476 (307.5, 673)
HIV-1 Viral Load (Log_10_ copies/mL)	4.8 (4.1, 5.4)	–	–	4.6 (3.7, 5.2)
Home-to-clinic distance (km)^a^				
0–4.9	36 (21.8)	36 (21.8)	58 (19.3)	58 (19.3)
5–9.9	55 (33.3)	55 (33.3)	114 (37.9)	114 (37.9)
10–14.9	46 (27.9)	46 (27.9)	77 (25.6)	77 (25.6)
≥15	28 (17.0)	28 (17.0)	52 (17.3)	52 (17.3)
Married to Study Partner	165 (98.2)	162 (97.0)	289 (96.0)	284 (94.7)
Number of Living Children				
0	13 (7.7)	18 (10.7)	42 (14.0)	37 (12.3)
1–2	81 (48.2)	86 (51.2)	164 (54.5)	180 (59.8)
≥3	74 (44.1)	64 (38.1)	95 (31.6)	84 (27.9)
Less than Primary school education	25 (14.9)	45 (26.8)	45 (15.0)	71 (23.6)
Informal Residence	85 (50.6)	83 (49.4)	153 (50.8)	161 (53.7)
Home Ownership/Rent				
Rent < KSh 2,000^b^	66 (39.3)	66 (39.3)	134 (44.5)	138 (45.9)
Rent ≥ KSh 2,000	87 (51.8)	87 (51.8)	140 (46.5)	140 (46.5)
Own home	15 (8.9)	15 (8.9)	27 (9.0)	23 (7.6)
Monthly household income (KSh)				
Couple does not earn income	20 (11.9)	20 (11.9)	35 (11.6)	35 (11.6)
Monthly income <8,000^c^	57 (33.9)	57 (33.9)	111 (36.9)	111 (36.9)
Monthly income ≥8,000	91 (54.2)	91 (54.2)	155 (51.5)	155 (51.5)

NOTE. IQR  =  interquartile range; KSh  =  Kenyan Shillings. Monthly household income is the sum of the income for both study partners.

a Linear distance, based on location of residence at enrollment. Data missing for 6 participants.

b Based on the median rent (2,000 KSh ≈ 26 USD).

c Based on the median monthly income (8,000 KSh ≈ 104 USD).

### Statistical Analysis

The outcome in this analysis was time until study follow-up interruption (FUI), defined as missing two consecutive quarterly study visits. We used Cox proportional hazards regression to evaluate the time from enrollment until the first FUI, calculated as the length of time between the date of the enrollment visit and the scheduled date of the first missed visit (three months after the last completed clinic visit). Participants were censored at the date for their last scheduled follow-up visit, or at the date of death for participants who died during follow-up. If the exact date of death was unknown, the censoring date was defined as three months after the last completed visit. The crude associations between FUI and factors of interest were estimated by hazard ratios (HR) and 95% confidence intervals (CI). We hypothesized *a priori* that HIV status would modify the associations between FUI and distance variables, and therefore all analyses were conducted separately for HIV-1-infected and HIV-1-uninfected individuals. For regression models, distance was considered both continuously and categorically. We assumed that distances beyond 5 kilometers (km) were likely to necessitate transport other than walking, and therefore categorized linear distance into 5 km intervals (0–5, 5–10, 10–15, and >15). Potential confounders were included stepwise in a multivariable model and remained in the model if the adjusted association differed from the unadjusted hazard ratio by more than 10% in either the HIV-1-infected or -uninfected model. Robust standard errors were used to relax the assumption of normally distributed standard errors. All statistical analyses were conducted in Stata 10.1 (StataCorp, College Station, TX) with statistical significance criteria set at α<0.05.

**Figure 1:**
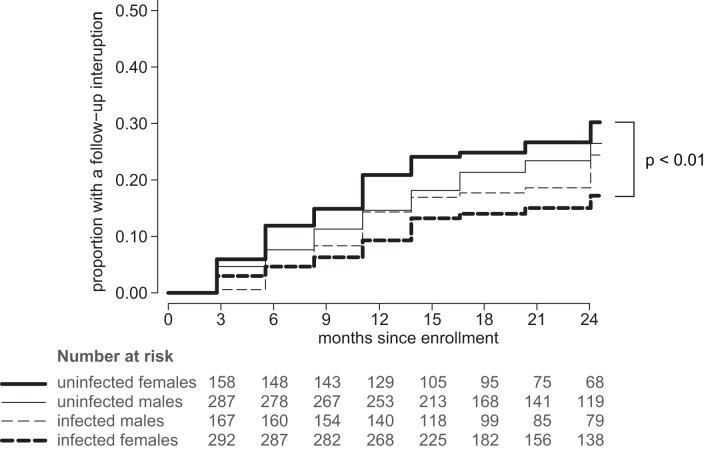
Cumulative incidence of follow-up interruptions. HIV-1-discordant couples were enrolled and followed quarterly for 2 years. A follow-up interruption (FUI) was defined as missing ≥2 consecutive study visits. The Kaplan Meier curves show the cumulative incidence of FUI separately for (**—**) HIV-1-uninfected females, (**———**), -infected females, (**—**) -uninfected males, and (– – –) -infected males.

## Results

### Study Population and Cohort Characteristics

Four hundred sixty nine HIV-1-discordant couples (938 individuals) were enrolled into the study. The male member of the couple was HIV-1-infected in 168 (36%) of enrolled couples, and the female was the infected partner in 301 (64%) of the couples. Median CD4 cell count at baseline in the HIV-1-infected was 415 cells/µl (Interquartile Range [IQR]: 281, 618) and median HIV-1 viral load was 4.6 log_10_ copies/ml (IQR: 3.9, 5.3). Among all enrolled, the median age at enrollment was 35 years (IQR: 30, 41) for men and 29 years (IQR 24, 34) for women (Table 1).

A total of 186 (20%) participants had less than a primary school education: 70 (15%) men and 116 (25%) women. Fifty-five (12%) of the couples enrolled reported no household income at study entry, 36% reported earning less than the median of 8,000 KSh per month, and 197 (52%) earned more than 8,000 KSh per month, equivalent to approximately 130 USD at the time of the study. Eighty (9%) participants reported owning a home at study entry with the remaining 858 (91%) renting their current residence.

**Figure 2:**
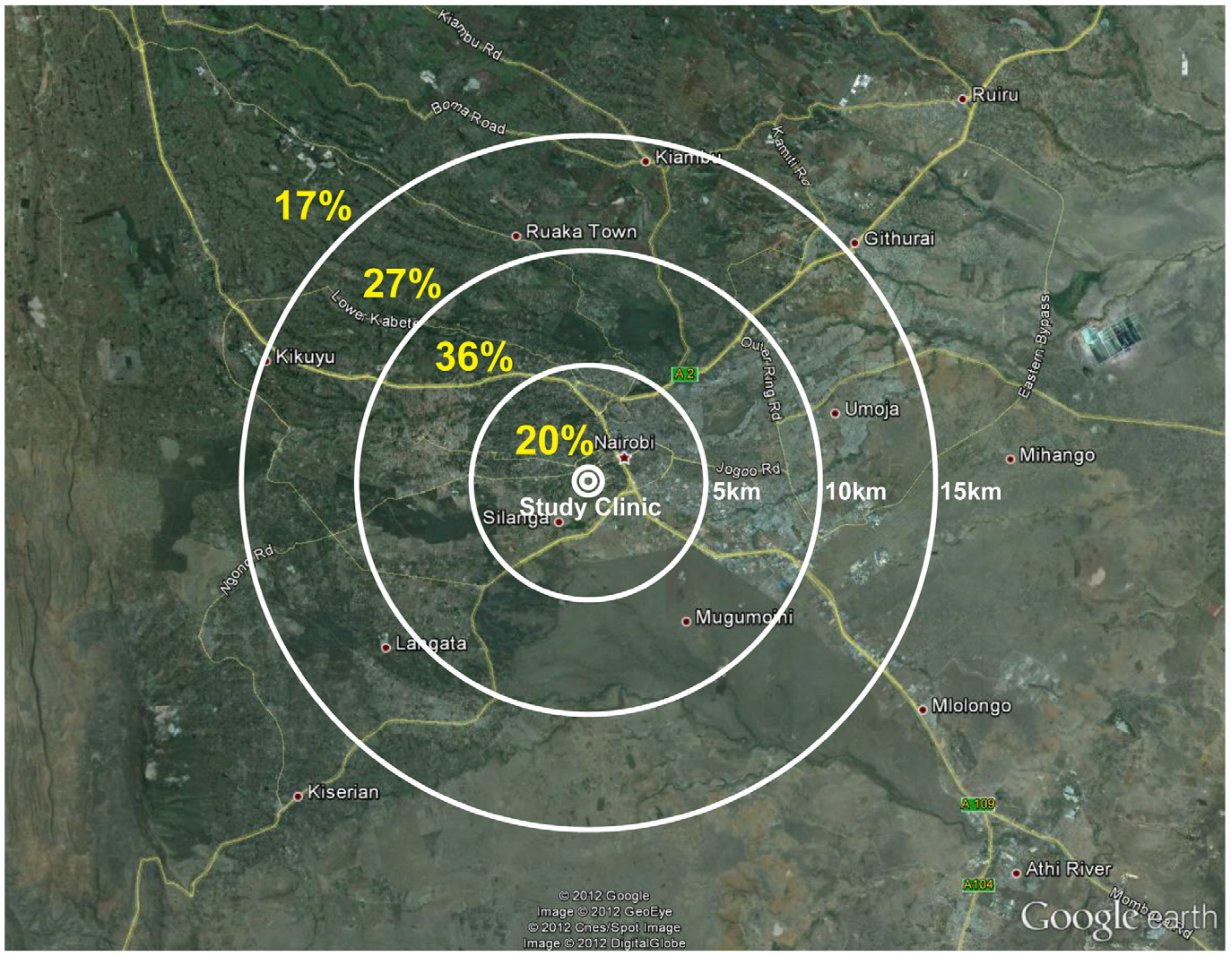
Map of distances from the study clinic. The map of Nairobi shows the location of the study clinic, with linear distances to the study clinic indicated by concentric white circles at 5 km increments. The percentage of participants living within each section is shown in yellow.

### Incidence of Follow-up Interruptions

Of those enrolled, 197 individuals missed two consecutive study visits and were categorized as having an FUI during 1471 person-years (PY) of follow-up, for a rate of 13.4 FUI per 100 PY. Among HIV-1-infected partners 46 women and 35 men experienced FUI and among HIV-1-uninfected partners, 46 women and 70 men experienced FUI (Figure 1). The rate of FUI was 10.8 per 100 PY in the HIV-1-infected and was significantly lower than the rate of 16.1 per 100 PY in the uninfected participants (HR = 0.66, p = 0.004). Among the HIV-1-infected, women had a slightly lower FUI rate than men (9.4 versus 13.2 per 100 PY; HR = 0.72, p = 0.138), while among the HIV-1-uninfected, women had a slightly higher FUI rate than men (18.2 versus 15.0 per 100 PY; HR = 1.22, p = 0.229). Among women, the HIV-1-infected had a significantly lower FUI rate than the uninfected (HR = 0.52, p<0.01). However, among the men, there was no difference in the FUI rate between HIV-1-infected and -uninfected (HR = 0.87, p = 0.485).

### Relationship between Home-to-Clinic Distance and FUI

For each couple we ascertained a linear distance between the residence and the study clinic (Figure 2). Patients lived a median of 9.14 km (IQR: 6.15, 12.79) from the clinic, with three couples living more than 100 km from the clinic. Eighty-three percent of couples lived within 15 km of the study clinic, and 20% of couples lived within 5 km.

Among HIV-1-infected participants, living between 5 and 10 km from the study clinic was significantly associated with a greater than two-fold increased likelihood of FUI when compared to those living <5 km from the clinic (Hazard Ratio [HR] 2.17; 95% CI: 1.09, 4.34; p = 0.03) (Table 2). Similar associations were seen in the 10–15 km (HR: 1.55, 95% CI: 0.74, 3.26; p = 0.25) and >15 km categories (HR: 1.48, 95% CI: 0.66, 3.33; p = 0.35), when compared to the less than 5 km distance category; however, these did not reach statistical significance. No significant associations between FUI and home-to-clinic distance were found among HIV-1-uninfected individuals. Similarly, no associations between FUI and home-to-clinic distance were found when distance was considered as a linear variable in the model.

**Table 2:** Correlates of study follow-up interruption.

	HIV-1-Infected		HIV-1-Uninfected	
Characteristic	HR	(95% CI)	HR	(95% CI)
Female gender	0.72	(0.47, 1.11)	1.26	(0.88, 1.81)
Age	1.00	(0.87, 1.15)	0.95	(0.84, 1.07)
Relationship duration	0.98	(0.94, 1.02)	0.98	(0.95, 1.01)
CD4 count	0.95	(0.88, 1.04)	–	–
HIV-1 viral load (Log_10_ copies/mL)	1.23 *	(1.01, 1.51)	–	–
Home-to-clinic distance (km)^a^				
0–4.9	1	ref	1	ref
5–9.9	2.17 *	(1.09, 4.34)	0.81	(0.50, 1.30)
10–14.9	1.55	(0.74, 3.26)	0.67	(0.39, 1.13)
≥15	1.48	(0.66, 3.33)	0.88	(0.50, 1.53)
Married to study partner	0.75	(0.27, 2.04)	0.53	(0.25, 1.10)
Number of living children				
0	1	ref	1	ref
1–2	0.61	(0.33, 1.15)	0.73	(0.44, 1.22)
≥3	0.62	(0.32, 1.20)	0.87	(0.51, 1.47)
Less than primary education	0.51 *	(0.27, 0.98)	1.34	(0.88, 2.04)
Informal residence	0.99	(0.65, 1.52)	1.22	(0.86, 1.75)
Own home/monthly rent				
Rent < KSh 2,000^b^	1	ref	1	ref
Rent ≥ KSh 2,000	1.67 *	(1.05, 2.65)	1.14	(0.78, 1.67)
Own home	1.23	(0.51, 2.97)	1.15	(0.61, 2.18)
Monthly household income (KSh)				
Couple does not earn income	1	ref	1	ref
Monthly income <8,000^c^	0.69	(0.36, 1.35)	0.68	(0.39, 1.19)
Monthly income ≥8,000	0.78	(0.42, 1.46)	0.64	(0.37, 1.09)

NOTE. HR  =  hazard ratio; CI  =  confidence interval; KSh  =  Kenyan Shillings. The HR for age is per 5 years, the HR for relationship duration is per 1 year, and the HR for CD4 count is per 100 cells/µl. Monthly household income is the sum of the income for both study partners. Confidence intervals were calculated using robust standard errors.

a Linear distance, based on location of residence at enrollment. Data missing for 6 participants.

b Based on the median rent. 2,000 KSh ≈ 26 USD.

c 8,000 KSh ≈ 104 USD.

* p<0.05.

To further investigate the finding that a residence 5–10 km from the study clinic was the highest risk group among HIV-1-infected participants, we conducted a series of post-hoc analyses. We found no significant difference in risk of FUI between HIV-1-infected participants living ≥10 km from the clinic compared to those living 5–10 km (HR = 0.70, 95% CI 0.44 to 1.11, p = 0.13), and when we collapsed these categories, there was an attenuated association between distance from study clinic and FUI (HR = 1.80 comparing ≥5 km to <5 km, 95% CI 0.94, 3.47, p = 0.08).

### Sociodemographic Correlates of Study Follow-up Interruption

We evaluated other sociodemographic factors as potential correlates of FUI using Cox regression with separate models for HIV-1-infected and -uninfected participants. Among HIV-1-infected participants, higher baseline log_10_ HIV-1 viral load was associated with increased FUI rates (HR = 1.23, 95% CI: 1.01, 1.51), and having less than a primary school education was associated with lower FUI rates (HR = 0.51, 95% CI: 0.27, 0.98). Also among HIV- infected individuals, paying greater than or equal to the median monthly rent (≥2000 KSh, approximately 26 USD) compared to rent less than the median rent was associated with a 67% increase in the rate of FUI (95% CI: 1.05, 2.65). We found no significant associations between baseline characteristics and FUI among HIV-1-uninfected participants. A multivariable model fit separately for HIV-1-infected and -uninfected patients including primary school education, monthly rent and log_10_ HIV-1 viral load (for the HIV-1 infected group only) found no independent associations with FUI (data not shown).

To determine whether gender modified the association between linear distance and FUI, we stratified the analysis by both gender and HIV-1 status. Among the HIV-1-positive women, living between 5 and 10 km from the study clinic was associated with a nearly three-fold increase in the FUI rate compared to those living <5 km from the clinic (95% CI: 1.01, 8.61; p = 0.05) (Table 3). Distance categories of 10–15 km and >15 km had increased FUI rates, but these did not achieve statistical significance (HR = 2.04, p = 0.22 and HR = 2.45, p = 0.14, respectively). Conversely, among HIV-1-uninfected males, there was a pattern of decreased FUI with home-to-clinic distances greater than 5 km, but these associations were not statistically significant. Overall we did not find that gender modified the association between distance and FUI; the likelihood ratio test for a distance/gender interaction was not significant in either HIV-1-infected (p = 0.420) or -uninfected groups (p = 0.999). Rent/home ownership was the only confounder in the association between distance and FUI. After adjustment for rent/home ownership, the 5–10 km distance was no longer statistically significant among the HIV-positive females.

**Table 3:** Association between home-to-clinic distance and follow-up interruption, by gender and HIV status.

	Couples with HIV-1-Infected Male Partner								Couples with HIV-1-Infected Female Partner							
	HIV-1-Infected Male				HIV-1-Uninfected Female				HIV-1-Uninfected Male				HIV-1-Infected Female			
	N = 168				N = 168				N = 301				N = 301			
Distancefrom Hometo Clinic (km)	Unadjusted		Adjusted^a^		Unadjusted		Adjusted^a^		Unadjusted		Adjusted^a^		Unadjusted		Adjusted^a^	
	HR	95%CI	HR	(95%CI)	HR	(95% CI)	HR	(95% CI)	HR	(95% CI)	HR	(95% CI)	HR	(95% CI)	HR	(95% CI)
**0 to <5**	1	ref	1	ref	1	ref	1	ref	1	ref	1	ref	1	ref	1	ref
**5 to <10**	1.74	(0.70, 4.30)	1.63	(0.67, 4.00)	1.01	(0.47, 2.18)	0.68	(0.37, 1.25)	0.72	(0.39, 1.31)	0.94	(0.41, 2.20)	2.95 *	(1.01, 8.61)	2.65	(0.88, 8.00)
**10 to <15**	1.21	(0.46, 3.20)	1.19	(0.46, 3.08)	0.56	(0.23, 1.39)	0.69	(0.35, 1.35)	0.74	(0.38, 1.41)	0.55	(0.22, 1.40)	2.04	(0.65, 6.38)	1.78	(0.54, 5.86)
**≥15**	0.83	(0.24, 2.82)	0.84	(0.63, 2.66)	1.16	(0.48, 2.78)	0.66	(0.29, 1.51)	0.74	(0.36, 1.52)	1.20	(0.50, 2.92)	2.45	(0.76, 7.89)	2.05	(0.62, 6.75)

NOTE. HR  =  hazard ratio; CI  =  confidence interval.

a Adjusted for housing status (own home, renting for less than the median, or renting for greater than or equal to the median).

* p<0.05.

## Discussion

In a longitudinal cohort of HIV-1-discordant couples followed quarterly, we found that rates of FUI were high (21% overall) and that distance between home and the study clinic was significantly associated with FUI, particularly among HIV-1-infected women. While HIV-1-infected female partners were the least likely to miss two consecutive quarterly visits, they were approximately three times more likely to experience FUI if they resided greater than walking distance (>5 km) from the clinic.

We found a complicated relationship between distance from participants’ residences and the study clinic and their likelihood of an FUI. The association between home-to-clinic distance and FUI was restricted to HIV-1-infected participants, and the strongest association was among those living 5–10 km from the study clinic with a weaker relationship among those living >10 km from the clinic. This pattern was stronger among HIV-1-infected women compared to infected men. Transportation challenges related to traveling more than 5 km to reach the clinic may pose a barrier to attending study visits. For example, those living within 5–10 km from the centrally located study clinic resided within city limits, potentially resulting in higher transportation costs (approximately 200 KSh per study visit) if they could not walk the distance to clinic. Participants living <5 km from the study clinic may have walked to study visits, or if not, were unlikely to have spent a large proportion of the compensation on direct transportation costs (likely less than 60 Ksh). Therefore, the 500 KSh per visit incentive, an amount equal to a quarter of the median monthly rent (2,000 KSh), may pose a greater incentive for those residing closer to the clinic compared to those living at or near the city limits. We did not find a significant association between longer home-to-clinic distances (i.e., >10 km) and the risk of FUI. However, the point estimates for this category were slightly lower than in the 5–10 km category, and the lack of statistical significance may simply represent a loss of statistical power. We hypothesize that those who lived a greater distance from the study clinic and chose to enroll may have a greater level of motivation to continue participation, possibly due to a desire to obtain specialized health care not available near their home or to avoid stigma related to HIV infection. Additionally, living farther from the center of town may be indicative of lower socioeconomic status and increased reliance on the financial reimbursement provided by the study, and thus decreased rates of FUI.

In this study, we found that women experienced fewer FUI than men, which is consistent with other studies that have found greater adherence to clinic visits and less program attrition among HIV-1-infected women compared to -infected men [8], [18], [23], [38]. It has been hypothesized that this is due to women’s motivation to remain healthy for her family [17]. While our study confirmed that HIV-1-infected women were less likely to miss study visits, we also found that within this group, home-to-clinic distance may play a role in FUI. HIV-1-infected women who are otherwise motivated to adhere to a study visit schedule may find the >5 km distance a challenge if they begin to experience HIV-related symptoms. A link between ill health and missed visits is supported by the fact that a higher viral load at enrollment was a significant predictor of increased risk of FUI. Higher viral load, a marker for HIV disease progression, could result in difficulty adhering to scheduled visits since participants who are ill may be unable to travel or may need to spend time seeking care and treatment rather than attending study visits. If antiretroviral treatment is commenced, participants with poor adherence to their medications may also demonstrate poor adherence to study visits as well. Additionally, previous studies have found that women spend more time traveling to obtain basic needs (e.g., food and water) for their families. Therefore, a longer distance to reach the study clinic may strain the physical and time resources of HIV-1-infected women compared to their male counterparts, resulting in the greater observed impact of distance on FUI among this group [39], [40].

We also found that paying higher rent was associated with a 67% increase in the FUI rate compared to paying lower rent. If a higher rent is an indicator of greater employment obligations, then the need to take time off from work to attend study visits may explain this association. However, since we saw no statistically significant association between household income and FUI, the association with rent may be influenced by other factors, such as the distance between the place of employment and study clinic. Having less than a primary education was also significantly associated with nearly half the rate of FUI compared to those with at least a primary education. Lower levels of education could limit a person’s ability to find stable employment, which could increase the need for the financial benefits of participating in a research study.

This study had several limitations. We were able to obtain measurements of the distance between participants’ residences and the clinic; however, we were unable to determine the time or financial cost required, or methods of transportation available. In one study, travel cost was found to be significantly associated with FUI, while the distance was not [41]; in another, travel time and distance were significantly associated, while cost was not [42]. Also, because this secondary study was conducted retrospectively, we were unable to assess participant perceptions of the impact of travel and distance on their ability to adhere to visits, or any other variables that could have affected study follow-up. In addition, we were only able to access locator information obtained at the first clinic visit. As a result, our data did not account for subsequent changes in residence.

Despite its limitations, our study provides important information for further investigation. We found that living further from the study clinic was not significantly associated with an increased risk of FUI, with the exception of HIV-1-infected women between 5 and 10 km, indicating that enrollment of participants living far from the clinic may not be inherently problematic. We also found that HIV-1-uninfected men and women had higher rates of interruption of study follow-up and are an important group on which to concentrate retention efforts in clinical trials and prospective studies that depend on retaining both HIV-1-infected and -uninfected participants. In this study, higher rates of FUI among HIV-1-uninfected partners may have been due to the lack of direct benefits from attending study visits. Unlike their infected partners who received CD4 cell count testing and drugs to prevent opportunistic infections at the study clinic, the uninfected partners may have experienced limited direct benefits from study participation, and consequently may have been less motivated to attend visits.

There has been limited research on retention of HIV-1-discordant couples in clinical research studies in sub-Saharan Africa. Yet these couples bear one of the greatest burdens of HIV transmission, and are already the focus of several large HIV prevention studies. Understanding and addressing transportation challenges in settings without easily accessible and inexpensive modes transportation could improve retention, both in research studies as well as in routine care and disease management. Studies combining quantitative and qualitative methods to address the barriers and incentives to study retention are needed to improve study retention and more importantly, to improve utilization and access to health care in sub-Saharan Africa settings.
